# Behavioral and Emotional Responding to Punishment in ADHD: Is Increased Emotionality Related to Altered Behavioral Responding?

**DOI:** 10.1007/s10802-024-01238-1

**Published:** 2024-08-31

**Authors:** An-Katrien Hulsbosch, Brent Alsop, Marina Danckaerts, Dagmar Van Liefferinge, Gail Tripp, Saskia Van der Oord

**Affiliations:** 1 Behavior, Health and Psychopathology, KU Leuven, Leuven, Belgium; 2 Child and Youth Institute, KU Leuven, Leuven, Belgium; 3 Human Developmental Neurobiology Unit, Okinawa Institute of Science and Technology Graduate University, Onna, Okinawa Japan; 4 Department of Psychology, University of Otago, Dunedin, New Zealand; 5 Center for Developmental Psychiatry, KU Leuven, Leuven, Belgium; 6 University Psychiatric Centre, KU Leuven, Leuven, Belgium; 7 Leuven Brain Institute, KU Leuven, Leuven, Belgium

**Keywords:** ADHD, Punishment, Response allocation, Emotional responding

## Abstract

Children with ADHD are theorized to experience increased negative emotional responses to punishment, compared to typically developing (TD) children, resulting in altered behavioral responding (Amsel, 1992). However, this has not been empirically tested. The current study evaluated the effects of punishment and reward on the behavioral and emotional responding of children with and without ADHD. Fifty-three children with ADHD (64.15% boys) and 46 TD children (47.83% boys), age 6–12, completed a task in which they chose between playing two simultaneously available games. Reward was arranged symmetrically across the games; responses on one game were punished four times as often as responses on the other game. Children’s negative and positive emotional expressions were assessed during task completion with facial expression coding. Results indicated both groups showed a preference for playing the less punished game. Children with ADHD took longer to respond after punishment and reward compared to TD children. Negative emotional expressions increased with time on task for those with ADHD, the opposite pattern was seen in TD children. Children with ADHD showed more positive emotional expressions overall. The effect of ADHD on increased response times after reward was statistically fully mediated by increased positive facial expressions. Findings indicate children with ADHD do not show an altered response bias under punishment compared to TD children, but their cumulative negative emotional responding may indicate problems with building frustration tolerance as hypothesized by Amsel (1992). Results are theoretically important as they suggest increased emotional responding in ADHD is associated with slower responding.

**Supplementary Information:**

The online version contains supplementary material available at 10.1007/s10802-024-01238-1.

## Introduction

Prominent theoretical accounts of attention-deficit/hyperactivity disorder (ADHD) assume altered reinforcement processing contributes to ADHD symptom development (Luman et al., 2010). Most of these theories predict altered sensitivity to positive reinforcement in ADHD (Sagvolden et al., 2005; Sonuga-Barke, 2003; Tripp & Wickens, 2008), and accumulating evidence exists to support this notion (e.g., Furukawa, 2022; Hulsbosch et al., 2021, 2023; Marx et al., 2021). Sensitivity to punishment on the other hand, has received limited attention in the field (Furukawa et al., 2017). This is somewhat surprising, as both positive (i.e., reward) and negative consequences (i.e., punishment) shape behavior and are used as behavior change techniques in daily life (van der Oord & Tripp, 2020). Two forms of punishment can be distinguished; negative punishment (i.e., response cost) where a rewarding stimulus is removed, and positive punishment, where an aversive consequence is delivered. Although multiple studies have investigated altered sensitivity to response cost in children with ADHD, only a small number of studies have considered the effects of positive punishment on the behavior or emotions of children with ADHD (but Furukawa et al., 2017, 2019).

To our knowledge, only three researchers have offered hypotheses regarding sensitivity to punishment in children with ADHD, with opposing views regarding the nature of its effects in children with and without ADHD. Wender (1975) and Quay (1997) assume children with ADHD show diminished sensitivity to punishment compared to typically developing (TD) children. Both propose this is caused by altered neurobiological functioning, but neither suggest specific mechanisms underlying such decreased sensitivity to punishment (Quay, 1997; Wender, 1975). In contrast, according to Amsel (1992), children with ADHD are more, rather than less, sensitive to punishment, as they experience it as more emotionally arousing (i.e., ‘frustrating’ according to Amsel) than TD children. Amsel does not distinguish between negative and positive punishment. He suggests that TD children develop ‘frustration tolerance’ and persist in goal-directed behavior under conditions of punishment, when the target behavior is sometimes rewarded, irrespective of the frustration evoked by the occurrence of punishment. Amsel (1992) hypothesizes children with ADHD have problems with developing such frustration tolerance as they experience higher levels of negative emotional arousal (i.e., ‘frustration’) in response to instances of punishment. Consequently, they do not show behavioral persistence under punishment conditions (Amsel, 1992). This notion of increased emotional responding to punishment is consistent with the idea that children with ADHD demonstrate heightened levels of emotional lability to negative events in general (Graziano & Garcia, 2016). Children with ADHD show more intense negative and positive emotions, and are more emotionally responsive to frustrative and negative situations (Graziano & Garcia, 2016).

Experimental evidence for the specific effects of punishment on the behavior of children with ADHD is mixed, with most studies focusing on the effects of response cost. In terms of behavioral responding, most available studies show improved performance in children with and without ADHD, across a wide range of tasks, under conditions of response cost (Crone et al., 2003; Cunningham & Knights, 1978; Drechsler et al., 2010; Firestone & Douglas, 1976; Groen et al., 2013b; Solanto, 1990; Van Meel et al., 2005a). In some studies, improved performance following response cost is only reported for children with ADHD, not TD children (Carlson et al., 2000; Carlson & Tamm, 2000; Iaboni et al., 1995; Slusarek et al., 2001), possibly due to ceiling effects in the performance of the latter group. Studies investigating responses to gambling tasks with both a reward and a penalty component (i.e., response cost) show increased risk-taking behavior in those with ADHD (Groen et al., 2013). Although multiple processes are likely operating in gambling tasks (e.g., altered sensitivity to reward, deficits in response inhibition), results of these tasks are sometimes interpreted as reduced punishment sensitivity as the possibility of a penalty does not reduce risk-taking. However, these findings cannot be easily disentangled from the effects of potential large rewards for risky choices.

Unlike altered sensitivity to negative punishment (i.e., response cost), only two studies have investigated the effects of positive punishment on the behavior of children with ADHD, showing increased rather than decreased sensitivity in children with ADHD (Furukawa et al., 2017, 2019). In these studies, children completed a response allocation task, in which they chose between playing two simultaneously available games, where the probability of being punished was higher for one game compared to the other. Different from existing gambling tasks, the availability of reward was symmetrical across the two response options. Punishment consisted of response cost and positive punishment being provided simultaneously. As the task was a response allocation task, increased sensitivity to punishment is assumed to result in a higher bias towards the less punished alternative. Both studies reported that children with ADHD demonstrated a larger bias towards playing the less punished alternative, an effect that was cumulative over task exposure, despite being associated with a poorer overall outcome (fewer points). Moreover, in both studies children with ADHD took longer to initiate a new trial after receiving a punishment or a reward compared to TD children (i.e., slower response times). Both results may be understood in terms of Amsel’s frustration theory, i.e., children with ADHD act to avoid the most punished alternative (and thus don’t show behavioral persistence), causing them to miss reward opportunities. The researchers suggested increased response times following punishment reflect increased emotional arousal, which is in line with Amsel’s notion of increased frustration in ADHD (Amsel, 1992). The increase in response times following rewarded responses in the children with ADHD might be explained by another theory; Douglas’s proposal that children with ADHD are more vulnerable to the arousing and distracting effects of reinforcement (Douglas, 1989).

A small number of studies have attempted to evaluate emotional responding to punishment at the neural level, using brain activity measurement techniques. One study using functional magnetic resonance imaging (fMRI) and two studies measuring event related potentials (ERP) showed increased neural activity to negative feedback in ADHD (Groen et al., 2013b; Van Dessel et al., 2022; Van Meel et al., 2005b). A third ERP study found decreased neural activity to punishment, but increased activity to the loss of anticipated reward in children with ADHD (van Meel et al., 2011). A final ERP study reported decreased neural activity to punishment in children with Inattentive, but not Combined presentation ADHD (Gong et al., 2014). Although these results offer some indications of altered emotional responding to punishment in children with ADHD, it is important to include other measurement methods (Mauss & Robinson, 2009). Emotion is a multidimensional construct, which cannot be captured by a single measurement method. Each method provides unique information relevant for the understanding of emotions (Mauss & Robinson, 2009).

Assessing emotions through behavioral measures is one such method and, although it has some limitations, is shown to be reliable in identifying the emotions an individual is experiencing (Jacob-Dazarola et al., 2016). There is some evidence of increased negative emotional responding in children with ADHD in response to frustrative events, using behavioral measures of emotional responding (e.g., Van Liefferinge et al., 2018; Wigal et al., 1998). A study of emotional responses to a task “unexpectedly” shutting down, reported more negative emotional expressions in children with ADHD compared to TD children (Van Liefferinge et al., 2018). In two other studies, children with ADHD showed increased negative emotional responding to frustrative non-reward, which was operationalized through speed of lever pulling (Douglas & Parry, 1994) or facial expression coding (Wigal et al., 1998). Additionally, in a paradigm assumed to measure frustration tolerance, children with ADHD showed decreased levels of frustration tolerance compared to TD children (Seymour et al., 2019). However, no studies to date have investigated the emotional responding of children with ADHD to punishment in an experimental task, allowing direct investigation of the relationship between behavioral and emotional responding.

Evaluating the effects of punishment on both behavioral and observed emotional responses will allow us to test Amsel’s predictions of increased frustration to punishment leading to altered behavioral responding. In the current study, we investigate the effects of punishment on both the behavioral (response allocation/median response times) and observable emotional responses (emotional expressions) of children with and without ADHD using the punishment paradigm described by Furukawa and colleagues (2017, 2019).

Consistent with previous findings with this task (Furukawa et al., 2017, 2019), we expected children with ADHD to show increased sensitivity to punishment, a cumulative effect, evidenced by the allocation of more responses (i.e., a bias) towards the less punished response alternative, compared to TD children. We also expected children with ADHD to take longer to initiate a new response following receipt of either reward or punishment. Given the hypothesized increased frustration to punishment experienced by children with ADHD (Amsel, 1992), and their generally higher levels of emotional lability (i.e., both more extreme negative and positive emotions; Graziano & Garcia, 2016), we expected children with ADHD to show increased emotional responding compared to TD children during the task. As both reward and punishment are delivered in the task, we expected increases in both positive and negative emotional responding in children with ADHD as compared to TD children (Amsel, 1992; Douglas, 1989). Finally, we expected the relationship between ADHD and altered behavioral responding (response bias/median response time) to punishment to be, at least partially, mediated through higher levels of negative emotional responding, given the assumed relation between these variables (Amsel, 1992). More precisely, we expect children with ADHD to show increased emotional responding, which is related to altered behavioral responding (i.e., increased response bias and increased response times after punishment). We expect similar (partial) mediating effects for positive emotional responding and increased response times after reward.

## Method

Ethical approval was obtained from the Social and Societal Ethics Committee, Faculty of Psychology and Educational Sciences at the KU Leuven, Belgium (G-2019 12 1922) and the Human Subjects Research Review Committee at the Okinawa Institute of Science and Technology (OIST) Graduate University, Japan (HSR-2021-003-2). Written consent was obtained from parents and teachers and children gave assent to participate in the study. The study was preregistered, and sample size (*n* = 84) was predetermined based on a power analysis. This power analysis was conducted in G*Power, considering the medium effect size (Cohen’s *d* = 0.54) as reported in the previous study of Furukawa et al. (2017), a power of 0.80 and α-value of 0.05 to detect possible main and interaction effects (see https://aspredicted.org/gi5ub.pdf).

### Participants

Data from fifty-three children meeting DSM-5 diagnostic criteria for ADHD (64.15% boys) and 46 TD children (47.83% boys), aged 6 to 12 years, was included in the study. Twenty children in the ADHD group met diagnostic criteria for the Inattentive presentation (37.74%), 5 for the Hyperactive/Impulsive presentation (9.43%) and 28 for Combined presentation (52.83%) ADHD. Children were recruited through regular schools and the clinical network of the authors in Belgium (ADHD: *n* = 19, TD: *n* = 36) or through an English language University ADHD Research Center in Japan (ADHD: *n* = 34, TD: *n* = 10). Children recruited in Japan were primarily US citizens and were native English speakers. All participants completed the task in a laboratory setting in a quiet, distraction-free room.

Participating children met the following inclusion criteria: (a) an (estimated) full scale IQ ≥ 70, measured with the Wechsler Intelligence Scale for Children-V (WISC-V; Matrix Reasoning and Vocabulary subtests for estimated IQ; Wechsler, 2014), (b) absence of a clinical diagnosis of autism spectrum disorder or any sensory or motor impairments or neurological condition as reported by the parents.

Children in the ADHD group from Belgium had a prior diagnosis of ADHD, established by a certified clinical psychologist or psychiatrist. This diagnosis was confirmed through a clinical and semi-structured parent diagnostic interview (Schedule for Affective Disorders and Schizophrenia for School-Age Children-Present and Lifetime version [K-SADS]; Kaufman et al., 2016) conducted by a Belgian licensed clinical psychologist. Children with ADHD tested in Japan completed a multimethod, multi-informant diagnostic assessment to determine if they met DSM-5 criteria for ADHD. This was based on a clinical and semi-structured parent diagnostic interview (K-SADS; Kaufman et al., 2016), parent and teacher ratings of ADHD symptoms from the Conners Behavioral Rating Scale (Conners, 2008b) and observations of the child’s behavior during the assessment. Diagnostic decisions were made by a US licensed clinical psychologist based on all available data. According to parent report, 1 child additionally met criteria for oppositional defiant disorder (ODD), 8 for a learning disorder and 4 for an anxiety disorder. Children with ADHD taking stimulant medication (*n* = 26) discontinued medication use at least 24 h prior to testing to allow for wash-out (Greenhill, 2002).

Typically developing children were required to show fewer than four symptoms of either inattention or hyperactivity/impulsivity as indicated by parent ratings of ADHD symptoms (*Belgium*: Disruptive Behavior Disorder Rating Scale [DBDRS]: Oosterlaan et al., 2008; *Japan*: Conners-3: Conners, 2008a). Different questionnaires were used based on the availability of the appropriate language and norms in the US/Belgium; both include the same DSM-5 items and use a similar 4-point rating scale.

### Experimental Task

**Fig. 1:**
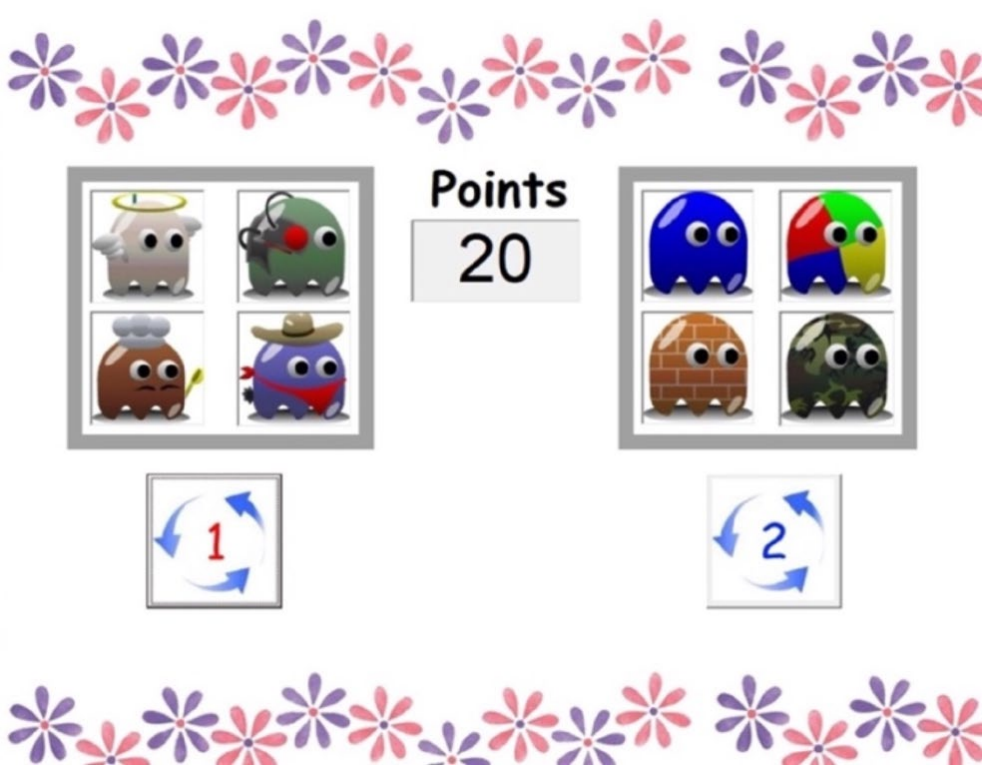
Appearance of the punishment task on the computer screen

The punishment task from Furukawa and colleagues (2017, 2019) was used in the current study. In this task, two games made up of 2 × 2 grids of cartoon characters, were presented on the left (Game 1) and right (Game 2) side of the computer screen, together with a center window showing accumulated points (see Fig. 1). A button below each game could be pressed to spin the cartoon characters for 3000-msec. If the four characters within one game were all the same when they stopped spinning (i.e., reward trial), the child received 10 points, a randomly selected animated cartoon played for 2500–3000-msec and there was a congratulatory “tada!” sound. When the trial resulted in four sad-faced characters (i.e., punishment trial), 5 points were taken from the child’s total (response cost) and a laughing sound “ha ha ha!” (positive punishment) played for 5000-msec. If the four characters did not match (i.e., non-consequential trial), there was no outcome. Once the child played one of the games, response buttons were disabled until the end of the trial and outcome delivery.

The availability of reward and punishment across both response options was based on the generalized matching law (Baum, 1974). According to this law, humans allocate their behavior in proportion to the rates of reinforcement available. Consequently, when reward is distributed symmetrically across response options, behavior is assumed to be equally distributed across the options as well. Superimposing asymmetric punishment on top of the symmetrical reward schedules allows to investigate the effects of punishment on the behavior of the participants, without the potential confounding of large rewards for risky choices. The availability of rewards was arranged equally across the two games (VI/VI) and became available every 10 s on average. For each block of 12 rewards delivered to the child, each game delivered 6, randomized across the block. As a result, each child received the same arranged distribution of rewards for both games in earlier and later trials. The first trial after switching games was never rewarded to avoid reinforcing switching behavior. Punishment was arranged asymmetrically across games where a response on Game 1 had a 16% probability of being punished, and Game 2 a 4% probability of punishment. Receiving reward or punishment on each game was the result of chance, and not based on a correct or incorrect action on the part of the child, thus no stimulus-response associations had to be learned. The rates of reward and punishment were chosen such that children would win more points than they would lose.

The task began with the instructions presented on the computer screen, that were read aloud by the experimenter. Children were told they could switch between playing the games as much as they like and if they won a lot of points, they would receive a prize at the end of the game. They started the task with a balance of 20 points. The task continued until the child reached a total of 400 points or completed 300 trials, whichever occurred first. The number of points/trials required to receive a prize was not disclosed to the child, as it might act to reduce rather than increase participant motivation. Completing the task took approximately 30-min and all children received a small prize after completion.

#### Data Extraction and Outcome Variables

The computer recorded the game chosen on each trial (Game 1 or Game 2), as well as the response time (from the time the response buttons became available) and the outcome of the trial (reward, punishment or non-consequential).

Response bias (log b) was calculated as follows:

$$\:\text{log}\,b=log10\,\frac{responses\:to\:Game\:2\:\left(less\:punished\right)}{responses\:to\:Game\:1\:\left(more\:punished\right)}$$

Scores above zero indicate a bias towards the less punished alternative. Response bias was calculated for the first and second blocks of 100 trials. Across all completed trials, median response times were calculated based on the outcome of the previous trial (reward, punishment or non-consequential).

### Emotional Expressions

#### Coding Procedure

Emotional expressions were coded for three 2-min blocks during the first 200 trials: at the beginning of the task, in the middle of the 200 trials (1-min before, and 1-min after the 100th trial) and before the 200th trial. Blocks were chosen to align with the blocks of trials used to calculate response bias, i.e., two blocks of 100 trials, to investigate the relationship between behavioral and emotional responding. An overview of the different outcome variables in relation to the task can be found in Supplementary Information S1.

Two coders with master’s degrees in clinical psychology were trained to code the children’s emotional expressions during these 2-min blocks (see Supplementary Information S2 for detailed explanation of the training procedure). One coder (primary coder) rated all recordings, the second coder (reliability coder) rated 20% of the recordings selected at random. The primary coder was aware of the child’s group status, whereas the reliability coder was blind to diagnosis.

#### Coding System

An adapted version of the facial expression coding system (FACES), which is a dimensional measure of facial behavior (Kring & Sloan, 2007), was used to code the children’s facial expressions. A facial expression was defined as a change from a neutral expression to a non-neutral expression and back to a neutral expression, or when a non-neutral expression changed into a different non-neutral expression. The valence of each facial expression was coded as positive (e.g., happy, surprised, etc.) or negative (e.g., sad, anxious, angry, etc.). The duration of the facial expression was coded when the expression exceeded 3-sec. When the duration of the expression exceeded 3-sec, each additional time-interval of 3-sec was coded into an additional expression in order to calculate the sum of all positive or negative facial expressions.

As research increasingly acknowledges the importance of measuring multiple modalities when measuring emotions through behavioral methods (Jacob-Dazarola et al., 2016), body movements and verbal expressions were coded as additional exploratory measures of emotional responding (See Supplementary Information S3). Percentage agreement based on Holsti’s method for all coded blocks (three for each participant) was high for facial expressions (88.89%), body movements (80.51%) and verbal expressions (87.5%).

#### Outcome Variables

The frequency of positive and negative facial expressions within each 2-min block was the main outcome measure for emotional expressions. This was calculated as the sum of all positive or negative facial expressions within that block, considering duration as described above. As an exploratory measure, total emotional expressions (i.e., the sum of facial expressions, body movements and verbal expressions), was calculated for positive and negative emotional expressions (see Supplementary Information S3 for full results).

### Statistical Analysis

Statistical analyses were performed using SPSS 28 (IBM, Armonk, NY). Demographic characteristics of the child (age, parent reported sex, estimated IQ) and ADHD symptom severity scores (i.e., the sum of raw parent-ratings on the DSM inattention and hyperactivity/impulsivity symptom items^1^) were compared between diagnostic groups (ADHD vs. TD) and test location (Belgium vs. Japan) using chi-square tests or two-way ANOVAs. Where significant differences were found, associations with the outcome variables were assessed to determine whether these characteristics would be controlled for in analyses of task performance and emotional expressions.

Inspection of the outcome variables for task performance and emotional expressions showed non-normal distributions for all outcome variables (high skewness and kurtosis). Log transformations were performed on the outcome variables (Field, 2009), with the exception of response bias as this outcome variable was already subjected to a log transformation. Data from two participants, one from each group, were excluded from the study as their response bias scores were identified as extreme outliers^2^. These participants were not included in the study sample. To investigate group differences in task performance, two repeated measures ANOVAs were conducted with group as between-subject variable. The within-subject variable depended on the outcome variable: block of 100 trials for response bias and outcome of the previous trial (reward, punishment and non-consequential) for median response time.

To investigate expected group differences in facial expressions, repeated measures ANOVAs were conducted with group as between-subject variable and the three 2-min blocks, for which facial expressions were coded, as the within-subject variable for positive and negative facial expressions. Partial eta squared effect sizes were calculated for all task performance and emotional expressions analyses (small effect: *η*_*p*_^*2*^ = 0.01, medium effect: *η*_*p*_^*2*^ = 0.06, large effect: *η*_*p*_^*2*^ = 0.014; Richardson, 2011).

Lastly, to investigate whether the relationship between ADHD and behavioral responding to punishment is (partially) mediated through increased emotional responding, several exploratory mediation analyses were performed. In these mediation analyses, group was included as the predictor, the number of facial expressions as the mediator and task performance as the dependent variable. Task performance outcome variables were only inserted in the mediation analyses when significant group differences on these variables were identified. The PROCESS script for SPSS was used for all mediation analyses (Hayes, 2022).

All analyses conducted on facial expressions were repeated for the exploratory measure of total emotional expressions. Results were largely in line with those of facial expressions, except when the contrary is indicated (see Supplementary Information S3 for full results).

## Results

### Demographic Characteristics and ADHD Symptom Severity

There was no difference between children with and without ADHD for parent-reported sex or age, and children tested in Belgium and US children living in Japan did not differ on these variables (see Table 1). No group differences were found for estimated IQ, but differences were found between children tested in Belgium and in Japan (*F*(1,95) = 9.23, *p* =.003, *η*_*p*_^*2*^ *=* 0.09). Post hoc analyses show no differences within the ADHD group (*F*(1,51) = 1.72, *p =*.196, *η*_*p*_^*2*^ = 0.03), but within the TD group, children recruited in Belgium had lower estimated IQs (*F*(1,44) = 8.44, *p* =.006, *η*_*p*_^*2*^ = 0.16; see Table 1). However, within the TD group, estimated IQ was not significantly correlated with any of the outcome variables and is thus not considered in further analyses.

**Table 1:** Demographic and clinical characteristics by diagnosis and test location

	ADHD		TD		Diagnosis F/χ² (*p*)	Location F/χ² (*p*)	Interaction F (*p*)^a^
	Belgium (*n* = 19)	Japan (*n* = 34)	Belgium (*n* = 36)	Japan (*n* = 10)			
Male sex, *n* (%)	16 (84.21)	18 (52.94)	19 (52.78)	3 (30.00)	2.67 (.102)	2.52 (.113)	-
Age (years), M (*SD*)	10.83 (1.11)	9.64 (1.93)	9.70 (1.57)	9.70 (1.62)	2.03 (.158)	2.51 (.116)	2.51 (.116)
Estimated FSIQ, M (*SD*)	104.21 (15.87)	109.21 (11.68)	102.44 (11.67)	115.50 (15.60)	0.58 (.448)	9.23 (.003**)	1.84 (.178)
ADHD symptom sum, M (*SD*)^b^							
Inattentive	17.37 (4.18)	15.54 (5.25)	3.75 (3.32)	5.15 (3.15)	153.62 (<.001***)	0.05 (.827)	2.77 (.099)
Hyperactive/impulsive	15.84 (4.05)	13.25 (5.85)	3.44 (3.01)	5.60 (3.43)	98.61 (<.001***)	0.05 (.829)	5.53 (.021*)
ADHD symptom count, M (*SD*)^c^							
Inattentive	6.79 (1.62)	5.53 (2.12)	0.50 (0.97)	0.50 (0.71)			
Hyperactive/Impulsive	6.16 (3.91)	3.91 (2.97)	0.47 (0.85)	1.00 (1.16)			
DSM-5 scales, T-score (SD)^d^							
Inattention scale		74.41 (10.75)		51.40 (7.83)			
Hyperactive/impulsive scale		73.47 (14.33)		54.60 (10.52)			

*Note* ADHD = Attention-deficit/hyperactivity disorder; FSIQ = Full-Scale IQ; SD = Standard deviation; TD = Typically developing

^a^ Interaction effects for Chi-square tests cannot be calculated

^b^ Measured with the ADHD rating scale (CBRS/Conners-3/DBDRS)

^c^ Number of symptoms endorsed on the semi-structured parent interview (children with ADHD; K-SADS) or ADHD questionnaire (TD children; DBDRS/Conners-3)

^d^ Measured with the CBRS or Conners-3

*p <.05, **p <.01, ***p <.001

The interaction between group and test location for parent ratings of ADHD symptom severity was significant for hyperactivity/impulsivity (*F*(1,95) = 5.53, *p* =.021, *η*_*p*_^*2*^ *=* 0.06), but not for inattention (*F*(1,95) = 2.77, *p* =.099, *η*_*p*_^*2*^ = 0.03). As expected, ADHD symptom severity scores were significantly higher in the ADHD group compared to the TD group for children tested in Belgium (Inattention: *F*(1,53) = 174.84, *p* <.001, *η*_*p*_^*2*^ = 0.77; Hyperactivity/Impulsivity: *F*(1,53) = 165.70, *p* <.001, *η*_*p*_^*2*^ = 0.76) and in Japan (Inattention: *F*(1,42) = 35.07, *p* <.001, *η*_*p*_^*2*^ = 0.46; Hyperactivity/Impulsivity: *F*(1,42) = 15.39, *p* <.001, *η*_*p*_^*2*^ = 0.27). Within the ADHD group, no differences were found between children in the two test locations (Inattention: *F*(1,51) = 1.69, *p* =.200, *η*_*p*_^*2*^ = 0.03; Hyperactivity/Impulsivity: *F*(1,51) = 2.94, *p* =.093, *η*_*p*_^*2*^ = 0.05). Similarly, within the TD group, there were no significant differences between children across test locations (Inattention: *F*(1,44) = 1.43, *p* =.239, *η*_*p*_^*2*^ = 0.03; Hyperactivity/Impulsivity: *F*(1,44) = 3.78 *p* =.058, *η*_*p*_^*2*^ = 0.08). Although a significant interaction emerged between test location and group on hyperactivity/impulsivity symptom severity, no difference between test locations within the ADHD group or TD group were found. Therefore, it is not considered further in the analyses.

### Task Performance

All children completed at least 200 trials, with the exception of one child who reached 400 points after 199 trials^3^. There was no difference between the two groups in the number of trials completed (*F*(1,97) = 1.58, *p* =.211, *η*_*p*_^*2*^ = 0.02). Amongst participants who completed all 300 trials, there was no significant difference between the two groups in the total accumulated points during the task (*F*(1,76) = 0.03, *p* =.859, *η*_*p*_^*2*^ = 0.00).

#### Response Bias toward the Less Frequently Punished Game

There was no significant main effect of group on response bias across the first and second block of 100 trials (*F*(1,97) = 0.42, *p* =.518, *η*_*p*_^*2*^ = 0.00). Neither the effect of block (*F*(1,97), = 2.24, *p* =.138, *η*_*p*_^*2*^ = 0.02), nor the interaction between group and block of 100 trials (*F*(1,97) = 0.59, *p* =.444, *η*_*p*_^*2*^ = 0.01) were significant. Children with and without ADHD did not differ in their response bias across the two blocks of 100 trials, and there was no difference between the first and second block of 100 trials (see Table 2). For both groups of children, the bias scores were significantly different from zero, i.e., they showed a significant bias toward the less punished response alternative for both blocks of 100 trials: ADHD group (first block of 100 trials: *t*(52) = 4.90, *p* <.001, *d* = 0.67; second block of 100 trials: *t*(52) = 4.40, *p* <.001, *d* = 0.61), TD group (first block of 100 trials: *t*(45) = 3.46, *p* <.001, *d* = 0.51; second block of 100 trials: *t*(45) = 3.75, *p* <.001, *d* = 0.55). For both groups, and across both blocks of 100 trials, reported effect sizes were medium in size (Cohen, 1988).

**Table 2:** Means and standard deviations for response bias towards the less punished game for each block of 100 trials and for the median response time (untransformed data) depending on each outcome type for both the ADHD and TD groups

	ADHD (*n* = 53)	TD (*n* = 46)
	*Bias for less punished game (log b), M (SD)*	
**Trial block**		
First block (Trial 1-100)	0.12 (0.18)	0.08 (0.16)
Second block (Trial 101–200)	0.14 (0.23)	0.14 (0.25)
	*Median response time, M (SD)*	
**Outcome type**		
Reward	2180.36 (1015.92)	1795.10 (660.34)
Punishment	2032.88 (799.16)	1776.01 (705.43)
Non-consequential	1239.53 (433.38)	1264.22 (404.73)

*Note* ADHD = Attention-deficit/hyperactivity disorder; M = Mean; SD = Standard deviation; TD = Typically developing

*p <.05, **p <.01, ***p <.001

#### Median Response Time Based on Outcome Type

There was a significant interaction effect between group and outcome type (i.e., reward, punishment, or non-consequential) for the median response times (*F*(2,194) = 4.29, *p* =.015, *η*_*p*_^*2*^ = 0.04), together with a main effect of outcome type (*F*(2,194) = 105.15, *p* <.001, *η*_*p*_^*2*^ = 0.52). The main effect of group on median response time was not significant (*F*(1,97) = 1.81, *p* =.182, *η*_*p*_^*2*^ = 0.02). Overall, children took longer to respond after a rewarded or a punished trial, compared to a non-consequential trial, an effect that was larger in children with ADHD compared to TD children (see Table 2). Response times following non-consequential trials were similar for both groups.

### Emotional Expressions

Video recordings for seven children (ADHD *n* = 4, TD *n* = 3) were missing due to technical difficulties (i.e., camera stopped recording during the task, or no recording was made). These children were excluded from the analyses involving emotional expressions.

Analysis of the number of negative facial expressions showed a significant interaction effect between the 2-min blocks and group (*F*(2,180) = 6.61, *p* =.002, *η*_*p*_^*2*^ = 0.07). The main effects of 2-min block (*F*(2,180) = 2.59, *p* =.078 *η*_*p*_^*2*^ = 0.03) and group (*F*(1,90) = 3.92, *p* =.051, *η*_*p*_^*2*^ = 0.04) were not significant. Children with ADHD showed significantly more negative facial expressions compared to TD children, but only during the last two blocks of 2-min for which emotional expressions were coded (see Table 3). The results for the exploratory measure of total number of negative emotional expressions, showed additional significant main effects of both group and 2-min block (see Supplementary Information S3).

**Table 3:** Means and standard deviations for the number of positive and negative facial emotional expressions (untransformed data) for each 2-min block for the ADHD and TD groups

	First block	Middle block	Last block
Negative facial expressions, M (SD)			
ADHD (*n* = 49)	5.65 (6.70)	8.22 (6.97)	8.47 (7.07)
TD (*n* = 43)	5.70 (5.57)	5.05 (5.26)	5.30 (5.85)
Positive facial expressions, M (SD)			
ADHD (*n* = 49)	5.53 (5.60)	3.90 (4.21)	3.63 (4.98)
TD (*n* = 43)	2.51 (2.55)	1.79 (2.92)	1.67 (2.33)

*Note* ADHD = Attention-deficit/hyperactivity disorder; M = Mean; SD = Standard deviation; TD = Typically developing

*p <.05, **p <.01, ***p <.001

For the number of positive facial expressions, both the main effect of 2-min block (*F*(2,180) = 9.78, *p* <.001, *η*_*p*_^*2*^ = 0.10) and group (*F*(1,90) = 9.12, *p* =.003, *η*_*p*_^*2*^ = 0.09) were significant. The interaction between 2-min block and group was not significant (*F*(2,180) = 0.57, *p* =.572, *η*_*p*_^*2*^ = 0.01). Children with ADHD showed significantly more positive facial expressions and the number of positive facial expressions decreased for both groups over the three 2-min blocks for which emotional expressions were coded (see Table 3).

### Mediating Effect of Emotional Expressions between ADHD and Task Performance

**Fig. 2:**
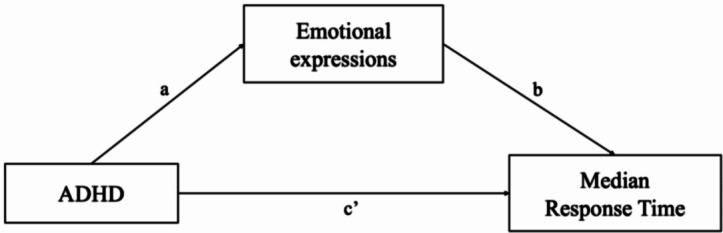
Visual representation of the investigated mediation model with negative/positive emotional expressions as a mediator in the relation between ADHD and median response time after punishment/reward

Given that children with and without ADHD only differed significantly on the median response times following punished and rewarded trials, the mediating role of facial expressions was only investigated for these outcome variables. However, median response times were calculated across all completed trials, but facial expression coding was done in three separate blocks of 2-min. Therefore, for these mediation analyses, the three 2-min blocks of facial expressions were summed into one measure, representing 6-min of facial expression coding. The mediating role of negative facial expressions was investigated in the effect of group on the median response time after punished trials; the mediating role of positive facial expressions in the effect of group on the median response time after rewarded trials (see Fig. 2).

#### Median Response Time After Punished Trials

As shown in Table 4, the total, indirect, and direct effects of group on median response time after punished trials were not significant for the model with negative facial expressions as mediator. Exploratory results on the total negative emotional expressions however, indicated the effect of group on median response time after punished trials was fully mediated by total negative emotional expressions (see Supplementary Information S3).

**Table 4:** Unstandardized coefficients with standard errors and indirect effects with 95% confidence interval (CI) for the different mediation models

	Response time after punishment		Response time after reward	
	Negative facial expressions		Positive facial expressions	
	b(SE)	t(*p*) / [95%CI]	b(SE)	t(*p*) / [95%CI]
a	0.13 (0.07)	1.99 (.049*)	0.25 (0.09)	2.71 (.008**)
b	0.10 (0.05)	1.77 (.081)	0.15 (0.04)	3.65 (<.001***)
c’ (direct effect)	0.04 (0.04)	1.05 (.297)	0.03 (0.04)	0.72 (.476)
a*b (indirect effect)^a^	0.01 (0.01)	[-0.01, 0.03]	0.04 (0.02)	[0.01, 0.07]*
c (total effect)	0.05 (0.03)	1.43 (.158)	0.06 (0.04)	1.68 (.097)

*Note* CI = confidence interval; SE = standard error

*p <.05, **p <.01, ***p <.001

^a^No p-values can be calculated for the indirect effects; * indicates the confidence interval does not contain the value 0 wherefore the indirect effect can be considered significant

#### Median Response Time After Rewarded Trials

The total and direct effects of group on median response time after rewarded trials were not significant for the model with positive facial expressions as mediator (see Table 4). However, the indirect effect of group on the median response time after rewarded trials via positive facial expressions (B = 0.04, SE = 0.02, 95% CI = [0.01, 0.07]) was found to be significant as the 95% CI did not include the value zero. Results thus indicate the relation between group and median response time after reward is fully mediated by positive facial expressions.

## Discussion

The behavioral and emotional responding of children with ADHD to punishment has received limited research attention despite its theoretical and clinical significance. To the best of our knowledge, the current study is the first to investigate the effects of positive punishment on both behavior (response allocation/median response time) and observed emotional responding in children with and without ADHD. The latter evaluated through coding of the children’s recorded facial expressions, as well as an exploratory measure of facial expressions, body movements and verbal expressions combined.

Contrary to our expectations, children with ADHD did not differ from TD children in their response bias toward the less punished alternative but did respond more slowly following both rewarded and punished trials. Notably, children with ADHD showed an increase in negative facial expressions over the course of the task, whereas this did not change in the TD sample. Children with ADHD expressed more positive facial expressions than TD children, with rates decreasing for both groups during the task. Higher rates of positive facial expressions mediated the relationship between ADHD and increased response times after reward. The relation between ADHD and increased response times after punishment, was not mediated by negative facial expressions.

The absence of significant group differences in the response bias scores toward the less punished alternative of children with and without ADHD differs from the results of earlier studies using the same punishment task (Furukawa et al., 2017, 2019). In both these earlier studies, children with ADHD showed a significant increase in response bias from the first to the second 100 trials. This effect was not seen in the children with ADHD in the current study. Thus, the current results do not support Amsel’s predictions, hypothesizing that children with ADHD show less behavioral persistence under punishment compared to TD children. Importantly, the response bias of both groups of children differed significantly from zero, confirming that the behavior of children with ADHD is at least as sensitive to the effects of punishment as that of TD children.

Lack of power does not explain the absence of significant differences in response bias between children with and without ADHD in the current study. Based on power analysis as conducted before the start of the study (see pre-registration), the current study is sufficiently powered. Effect sizes as calculated from both previous studies were medium (Cohen’s *d* = 0.54; Furukawa et al., 2017) to large (Cohen’s *d* = 1.20; Furukawa et al., 2019), whereas current effect sizes were small in magnitude. Other sample characteristics may offer an explanation for the lack of significant results in response bias, such as differences in comorbidity rates, socio-economic status, or cultural background, and can be investigated in future studies.

Despite the absence of group differences in response bias, response times following punishment were significantly slower for children in the ADHD group, suggesting children with ADHD may be more sensitive to, or at least aware of, punishment than TD children. It has been suggested this increased response time may reflect heightened emotional arousal in response to punishment in the ADHD group (Furukawa et al., 2017). The negative emotional expression data from the current study is consistent with this interpretation. A significant increase in the frequency of negative facial expressions over the course of the task was observed in the ADHD but not the TD group, suggesting the negative emotional effects of punishment may be cumulative in ADHD. Amsel (1992) suggests children with ADHD have problems building frustration tolerance; our findings of cumulative negative emotional responding may be an indication of such increased negative emotional arousal. Typically developing children showed a decrease in negative facial expressions over the course of the task, potentially indicating the development of frustration tolerance. These findings are consistent with previous findings showing that children with ADHD show more negative emotional expressions in response to frustrative tasks (Van Liefferinge et al., 2018), instances of unexpected non-reward (Douglas & Parry, 1994; Wigal et al., 1998), and to stressful and frustrating situations (Graziano & Garcia, 2016).

The mediation analyses showed the effect of ADHD on median response time following punishment was not mediated by negative facial expressions. This argues against Amsel’s hypothesis that the increased frustration as found in children with ADHD to punishment, leads to altered behavioral responding (Amsel, 1992). This mediation did become significant when total emotional expressions was inserted as mediator. We included this more comprehensive measure of emotional responding in response to recommendations of the field to include multiple modalities when coding emotional expressions (Jacob-Dazarola et al., 2016). However, in the current study, we treat it as more exploratory, as it is less well validated than facial expressions alone, and future studies should be conducted to increase the validity of this measure.

The increased response times and heightened emotional responding was not specific to punishment; children with ADHD also responded more slowly than TD children following rewarded trials. They also evidenced more positive facial expressions than TD children overall, which fully mediated the relationship between ADHD and increased response times after rewarded trials. These results are consistent with Douglas’s predictions that children with ADHD experience reinforcement as more arousing and distracting (Douglas, 1989), and with findings of increased arousal and more positive emotional reactivity in children with ADHD (Graziano & Garcia, 2016; McKay et al., 2023).

Although the children with ADHD in the current study did not show a cumulative response bias towards the less punished alternative, the results of median response times and negative facial expressions generally support the hypothesis of increased sensitivity to punishment as predicted by Amsel (1992) and argue against the proposed decreased sensitivity to punishment (Quay, 1997; Wender, 1975). Results also indicate that being exposed to punishment is associated with a significant increase in negative facial expressions over time in children with ADHD, suggesting punishment may have negative effects on the emotional well-being of children with ADHD. The findings also indicate children with ADHD show increased observable emotional responding to motivationally significant events in general, i.e., both reward and punishment. This supports Douglas’s hypothesis of increased arousal in children with ADHD, following reinforcement (Douglas, 1989). Together, these results highlight the importance of considering emotional responses to experimental tasks beyond task parameters assessing behavioral responding (e.g., task performance, behavioral allocation, response speed, etc.) to reward and/or punishment. The higher levels of emotional expressions in children with ADHD also supports the suggestion that increased emotional reactivity should be considered as an important process contributing to ADHD symptomatology and may be related to some of its underlying processes, such as altered reinforcement sensitivity. Additionally, it is also in line with the notion that increased emotional reactivity should be part of the assessment of ADHD (Barkley, 2015).

The current study is not without limitations. Data collection was conducted in two countries: Belgium and Japan (US citizens). Within the TD group, children from Belgium had lower estimated IQ scores. However, estimated IQ was not related to any of the outcome variables within the TD group. Although both positive and negative emotional expressions were coded over multiple 2-min blocks during the task, emotional reactivity in response to *individual* occurrences of reward or punishment was not evaluated. Both positive punishment and response cost were delivered simultaneously on punished trials. As a result, the effects of positive and negative punishment cannot be separated in the current study. While behavioral measurement of emotions through behavioral coding are shown to be valid and reliable (Jacob-Dazarola et al., 2016), emotions cannot be fully captured by a single measurement method. Future studies should include additional measures of emotional responding, such as self-report or physiological measures (e.g., heart rate variability or skin conductance) of emotions (Mauss & Robinson, 2009). The primary coder for the emotional expression coding was not blind for the participants’ ADHD diagnosis, raising the possibility that the emotional expression ratings might be biased. However, the reliability coder was blind to the children’s group membership and presumably unbiased. Interrater agreement between the raters was high, suggesting that both raters were coding emotional expressions similarly. Finally, in his theory, Amsel assumes increased frustration in children with ADHD (Amsel, 1992). However, what is considered “frustration” is not made clear in his theory and our measure of negative emotional expressions might be a more general or broader measure of negative emotional processes than the emotional response predicted by the theory.

The findings of the current study, if replicated, are potentially relevant for clinical and educational practices, as they suggest children with ADHD take longer to initiate a new response following reward and punishment compared to their TD peers and show increased emotional responding. This may translate to daily life where parents and/or teachers deliver reward and punishment. First, parents and teachers might be well advised to provide children with ADHD more time to process the consequences of their actions. Second, research shows increased emotional responding of children with ADHD can have a significant negative effect on the efficiency of psychosocial interventions (Bagner et al., 2012; Gatzke-Kopp et al., 2015). Providing the children with ADHD and their parents with emotion regulation strategies may be a valuable addition to psychosocial interventions, as supported by previous research (Rosen et al., 2019).

## Electronic Supplementary Material

Below is the link to the electronic supplementary material.

Supplementary Material 1

Supplementary Material 2

Supplementary Material 3

## Data Availability

Data are available upon request to the first author.

## Footnotes

1 Some DSM symptoms are represented by two items of the CBRS/Conners 3. For these symptoms, the mean of the two items was recorded.

2 Extreme outliers were defined as more than three box lengths from the lower or upper hinge of the boxplot.

3 Data from this child was included in all analyses. Response bias for the second block of 100 trials was calculated for 99 trials instead of 100. The last 2-min block of emotional expressions was coded before the 199th trial instead of the 200th trial.
