# Supplementary material for: Behavioral and Emotional Responding to Punishment in ADHD: Is Increased Emotionality Related to Altered Behavioral Responding?
Source: Res Child Adolesc Psychopathol. 2024 Aug 31;52(12):1817–29. doi: 10.1007/s10802-024-01238-1 (PMC11624220; doi:10.1007/s10802-024-01238-1)
Supplement: Supplementary file 1 — Supplementary Material 1 [file 10802_2024_1238_MOESM1_ESM.docx]

**Title:** Behavioral and emotional responding to punishment in ADHD: is increased emotionality related to altered behavioral responding?

**Journal name**: Research on Child and Adolescent Psychopathology

**Author names:** An-Katrien Hulsbosch, Brent Alsop, Marina Danckaerts, Dagmar Van Liefferinge, Gail Tripp* & Saskia Van der Oord* (*joint last authors)

**Corresponding author:** An-Katrien Hulsbosch

**Fig S1.** Overview of the different outcome variables in relation to the task structure
