# Supplementary material for: Behavioral and Emotional Responding to Punishment in ADHD: Is Increased Emotionality Related to Altered Behavioral Responding?
Source: Res Child Adolesc Psychopathol. 2024 Aug 31;52(12):1817–29. doi: 10.1007/s10802-024-01238-1 (PMC11624220; doi:10.1007/s10802-024-01238-1)
Supplement: Supplementary file 2 — Supplementary Material 2 [file 10802_2024_1238_MOESM2_ESM.docx]

**Title:** Behavioral and emotional responding to punishment in ADHD: is increased emotionality related to altered behavioral responding?

**Journal name**: Research on Child and Adolescent Psychopathology

**Author names:** An-Katrien Hulsbosch, Brent Alsop, Marina Danckaerts, Dagmar Van Liefferinge, Gail Tripp* & Saskia Van der Oord* (*joint last authors)

**Corresponding author:** An-Katrien Hulsbosch

**Supplementary Information S2**

**Training procedure for the emotional expression coding system**

Training was undertaken with recordings of children who did not meet the studies inclusion criteria. During the first training session the coding manual and coding sheets were discussed, a first practice recording was coded, and questions were resolved. After this first training session, each coder coded two videos for which discrepancies were discussed and further questions were answered. Next, sets of two videotapes were coded separately for which percentage agreement was calculated based on Holsti’s method, allowing for an unequal number of expressions coded for each coder (Mao, 2017). Practice coding in blocks of two videos was repeated until percentage agreement of 80% was achieved after which coding of included participants started.
