# Supplementary material for: Behavioral and Emotional Responding to Punishment in ADHD: Is Increased Emotionality Related to Altered Behavioral Responding?
Source: Res Child Adolesc Psychopathol. 2024 Aug 31;52(12):1817–29. doi: 10.1007/s10802-024-01238-1 (PMC11624220; doi:10.1007/s10802-024-01238-1)
Supplement: Supplementary file 3 — Supplementary Material 3 [file 10802_2024_1238_MOESM3_ESM.docx]

**Title:** Behavioral and emotional responding to punishment in ADHD: is increased emotionality related to altered behavioral responding?

**Journal name**: Research on Child and Adolescent Psychopathology

**Author names:** An-Katrien Hulsbosch, Brent Alsop, Marina Danckaerts, Dagmar Van Liefferinge, Gail Tripp* & Saskia Van der Oord* (*joint last authors)

**Corresponding author:** An-Katrien Hulsbosch

**Supplementary Information S3**

**Results for the exploratory measure of total emotional expressions**

Body movements and verbal expressions were coded as additional exploratory measure of emotional responding but were only coded if the valence of the expression was clear and unambiguous (i.e., clearly positive or negative; see Table S1 for examples). The valence of these expressions was coded, as well as the duration when they exceeded 3 sec (except verbal expressions which were considered as a single expression, e.g., full sentences lasting longer than 3 sec). When the duration exceeded 3 sec, additional time-intervals of 3 sec were coded into an additional expression to calculate the sum of all expressions. The total number of emotional expressions, which is the sum of all facial expressions, body movements and verbal expressions, was calculated for each 2-min block as the outcome measure, separately for positive and negative expressions.

**Table S1. Examples of negative, positive, and ambiguous body movements and verbal expressions**

| **Body movements** | | |
| --- | --- | --- |
| Negative | Positive | Ambiguous |
| Slamming table with hands | Throwing hands in the air to celebrate | Resting head on hands |
| Covering eyes with hands | Victory dance | Fidgeting with hair (or other things) |
| **Verbal expressions** | | |
| Negative | Positive | Ambiguous |
| No don’t take my points! | Yes, I got points! | If I play this game, I have more luck! |
| This is a stupid game! | Laughing/giggling | Did you develop this game? |

**Emotional expressions**

For the total number of negative emotional expressions (i.e., the sum of negative facial expressions, verbal expressions and body movements), the interaction effect between 2-min block and group was significant (*F*(2,180) = 8.16, *p* < .001, *η_p_^2^* = .083), as well as the main effect of 2-min block (*F*(2,180) = 5.95, *p* = .003, *η_p_^2^* = .062) and group (*F*(1,90) = 7.38, *p* = .008, *η_p_^2^* = .076). Children with ADHD showed more negative emotional expressions compared to TD children, but only in the last two blocks of 2-min for which emotional expressions were coded (see Table S1).

The results for the frequency of total positive emotional expressions (positive facial expressions, verbal expressions and body movements combined) showed both a significant main effect of 2-min block (*F*(2,180) = 5.74, *p* = .004, *η_p_^2^* = .060) and a significant main effect of group (*F*(1,90) = 12.12, *p* < .001, *η_p_^2^* = .119). The interaction effect between 2-min block and group was not significant (*F*(2,180) = 0.04, *p* = .960, *η_p_^2^* = .000). All children showed a decline in the number of positive emotional expressions over the 2-min blocks and children with ADHD showed more positive expressions compared to TD children in all 2-min blocks for which emotional expressions were coded (see Table S1).

**Mediating effect of emotional expressions between ADHD and task performance.**

**Median response time after punished trials.** As shown in Table S2, both the total and direct effects of group on median response time after punished trials were not significant for the model with total negative emotional expressions. However, the indirect effect of group on median response time after punished trials via total negative emotional expressions was found to be significant, as the 95% confidence interval (CI) did not contain the value zero (B = 0.03, SE = 0.01, 95% CI = [0.005, 0.05]). These results indicate the relationship between group and median response time after punished trials was fully mediated by the total negative emotional expressions.

**Median response time after rewarded trials.** Both the total and direct effects of group on median response time after rewarded trials were not significant for the model with total positive emotional expressions as mediator (see Table S2). However, the indirect effect of group on the median response time after rewarded trials via total positive emotional expressions was found to be significant (B = 0.05, SE = 0.02, 95% CI = [0.01, 0.09]) as the 95% CI did not include the value zero. Results thus indicate the relation between group and median response time after reward is fully mediated by total positive emotional expressions.

Table S1. Means and standard deviations for the number of positive and negative total emotional expressions (untransformed data) for each 2-min block for the ADHD and TD groups.

|  | First block | Middle block | Last block | Diagnosis  *F* (*p*) | Block  *F* (*p*) | Interaction  *F* (*p*) |
| --- | --- | --- | --- | --- | --- | --- |
| Total negative expressions |  |  |  |  |  |  |
| ADHD (*n* = 49) | 7.96 (7.91) | 15.31 (12.81) | 15.18 (15.60) | 7.38 (.008**) | 5.95 (.003**) | 8.16 (<.001***) |
| TD (*n* = 43) | 7.35 (7.87) | 7.42 (8.39) | 8.49 (9.91) |  |  |  |
| Total positive expressions |  |  |  |  |  |  |
| ADHD (*n* = 49) | 8.00 (7.87) | 6.63 (7.61) | 6.71 (10.30) | 12.12 (<.001***) | 5.74 (.004**) | 0.04 (.960) |
| TD (*n* = 43) | 3.56 (4.12) | 3.09 (6.54) | 2.42 (3.67) |  |  |  |

*Note.* ADHD = Attention-deficit/hyperactivity disorder; M = Mean; SD = Standard deviation; TD = Typically developing.

**p* < .05, ***p* < .01, ****p* < .001

Table S2. Unstandardized coefficients with standard errors and indirect effects with 95% confidence interval (CI) for the mediation models with total emotional expressions.

|  | Response time after punishment | | Response time after reward | |
| --- | --- | --- | --- | --- |
|  | Total negative expressions | | Total positive expressions | |
|  | β(SE) | β(SE) | β(SE) | t(*p*) / [95%CI] |
| a | 0.23 (0.08) | 0.34 (0.10) | 0.34 (0.10) | 2.90 (.005**) |
| b | 0.11 (0.05) | 0.14 (0.04) | 0.14 (0.04) | 1.39 (.167) |
| c' (direct effect) | 0.02 (0.04) | 0.02 (0.04) | 0.02 (0.04) | 1.20 (.232) |
| a*b (indirect effect)^a^ | 0.03 (0.01) | 0.05 (0.02) | 0.05 (0.02) | [-0.01, 0.04] |
| c (total effect) | 0.05 (0.03) | 0.06 (0.04) | 0.06 (0.04) | 1.68 (.097) |

*Note.* CI = confidence interval; SE = standard error.

**p* < .05, ***p* < .01, ****p* < .001

^a^No p-values can be calculated for the indirect effects; * indicates the confidence interval does not contain the value 0 wherefore the indirect effect can be considered significant.
